# Retraction Note: Microwave-ultrasonic assisted extraction of lignin to synthesize new nano micellar organometallic surfactants for refining oily wastewater

**DOI:** 10.1186/s40643-024-00819-8

**Published:** 2024-10-18

**Authors:** M. H. Alhalafi, S. A. Rizk, E. S. Al-Malki, A. M. Algohary

**Affiliations:** 1 Department of Chemistry, Faculty of Science Al-Zulfi, Majmaah University, 11952 Majmaah, Saudi Arabia; 2 Department of Chemistry, Science Faculty, Ain Shams University, Cairo, 11566 Egypt; 3 Department of Biology, College of Science Al-Zulfi, Majmaah University, 11952 Majmaah, Saudi Arabia; 4 Egyptian Drug Authority (EDA), P.O.29, Giza, Egypt

**Retraction Note: Bioresources and Bioprocessing (2024) 11:46**

10.1186/s40643-024-00761-9

The Editors-in-Chief have retracted this article because there is substantial overlap of text and data with a previously published article (Alsabagh et al., 2023). This article is therefore redundant. S.A. Rizk, H. Alhalafi, E. S. Al-Malki and A. M. Algohary do not agree with this retraction.
